# Supplementary material for: Longitudinal Assessment of Multimorbidity Medication Patterns among Smokers in the COPDGene Cohort
Source: Medicina (Kaunas). 2023 May 18;59(5):976. doi: 10.3390/medicina59050976 (PMC10221198; doi:10.3390/medicina59050976)
Supplement: Supplementary file 1 [file medicina-59-00976-s001.zip › medicina-2315879-supplementary.pdf]

Table S1 A total of 27 classes medication classification and generic names shared in both P1 and P2

Medication classification	Generic names
**ACEi**	captopril, enalapril, fosinopril, lisinopril, tensopril, hipril, perindopril, quinapril, accupril, ramipril, trandolapril, benazepril
**Antiplatelet**	aspirin, clopidogrel, prasugrel, ticagrelor, ticlopidine, cilostazol
**ARB**	candesartan, eprosartan, irbesartan, losartan, omesartan, telmisartan, valsartan
**Anxiolytic-hypnotics**	zolpidem, hydroxyzine, lorazepam, diphenhydramine, buspirone, flurazepam, triazolam, clonazepam, chlordiazepoxide, eszopicolone, meprobamate, chloral hydrate, ethchlorvynol, temazepam, secobarbital, oxazepam, zalepian, chlorazepate, diazepam, alprazolam, midzolam
**Alpha and Beta**	carvedilol, labetolol
**Biguanides**	metformin
**Beta blockers**	acebutolol, atenolol, betaxolol, bisoprolol fumarate, carteolol hydrochloride, metoprolol tartrate, metoprolol succinate, nadolol, penbutolol sulfate, pindolol, propranolol hydrochloride, solotol hydrochloride, timolol maleate
**Ca Channel blockers**	amlodipine, bepridil, diltiazem hydrochloride, felodipine, isradipine, nicardipine, nifedipine, nisoldipine, verapamil
**Central agonist**	alpha methyldopa, clonidine, guanabenz, guanfacine
**Diuretic**	chlorthalidone, chlorothiazide, furosemide, hydrochlorothiazide, indapamide, metolazone, amiloride hydrochloride, spironolactone, triamterene, bumetanide, moduretic, aldactazide, dyazide, maxzide
**DRI**	bupropion
**Fibrates**	lopid, fenofibrate, clofibrate
**Gout medication**	probenecid, allopurinol, febuxostat, colchicine, sulfinpyrazone, pegloticase
**H2 blocker**	cimetidine, ranitidine, famotidine, nizatidine
**Other Antidepressants**	isocarboxazid, phenelzine, tranylcypromine, trazodone, nefazodone
**Other hypertension**	doxazosin, prazosin, terazosin, carvedilol, labetolol, guanadrel, guanethidine, reserpine
**Other diabetic**	exenatide, liraglutide, vildagliptin, sitagliptin, saxagliptin, linagliptin, repaglinide, nateglinide, miglitol, acarbose, pramlintide, insulin
**PPI**	esomeprazole, lansoprazole, pantoprazole, omeprazole, rabeprazole
**Prostaglandin**	misoprostol
**SNRI**	duloxetine, venlafaxine, levomilnacipran, desvenlafaxine, milacipran
**SSRI**	citalopram, escitalopram, fluvoxamine, paroxetine, fluoxetine, sertraline, olanzapine and fluxetine hci
**Sulfonylureas**	tolbutamide, acetohexamide, tolazamide, chlorpropamide, glipizide, glyburide, glibenclamide, glimepiride, gliclazide

**Statin**	atorvastatin, Fluvastatin, lovastatin, pravastatin, rosuvastatin, simvastatin, niacin and lovastatin, atorvastatin and amlodipine, ezetimibe and simvastatin
**Selective cholesterol absorption inhibitors**	ezetimibe
**Tricyclic antidepressant**	clomipramine, amoxapine, amitriptyline, desipramine, nortriptyline, doxepine, trimipramine, imipramine, protriptyline
**Thiazolidinediones**	rosiglitazone, pioglitazone
**Vasodilator**	hydralazine, minoxidil

ACEi: angiotensin-converting enzyme inhibitors, ARB: angiotensin II receptor blockers, DRI: dopamine reuptake inhibitor, PPI: proton pump inhibitor, SNRI: serotonin and norepinephrine reuptake inhibitors, SSRI: selective serotonin reuptake inhibitors, P1: phase 1, P2: phase 2 (5-year follow up).

Table S2 Comparison of constrained and non-constrained latent transition model

	**Constrained model** [a]	**G^2**	**AIC**	**BIC**	**G^2_Δ**	**DF_Δ**	***P*-value** [b]
**4-solution**	Yes	48453.64	48699.6	49514.4	963.86	108	<0.0001
	No	47489.78	47951.8	49481.94			

AIC: Akaike Information Criterion, BIC: Bayesian information criterion, DF: degree of freedom, P1: phase 1, P2: phase 2 (5-year follow up).

a. Measurement invariance is not achieved because the comparison between constrained and non-constrained model was statistically significant (*P*<0.05).

b. *P*-value was calculated through likelihood-ratio test.

Table S3 Comparison of medication patterns to self-report morbidities at P1 and P2 separately

	**Low medication**	**HTN/CVD+Hychol predominant**			**HTN/CVD+T2D+Hychol predominant**			**Hychol predominant**		
	**n (%)**	**n (%)**	**OR** [a]	**95% CI**	**n (%)**	**OR** [a]	**95% CI**	**n (%)**	**OR** [a]	**95% CI**
**P1**										
**T2D**	162 (5.23)	163 (23.62)	5.61	4.43, 7.10	**239 (93.73)**	270.87[b]	159.44, 460.18	133 (8.98)	1.79	1.41, 2.27
**HTN**	662 (21.10)	**600 (86.96)**	24.94[b]	19.66, 31.62	**181 (70.98)**	9.15	6.89, 12.15	**931 (62.86)**	6.34	5.54, 7.27
**Stroke**	47 (1.50)	33 (4.78)	3.30	2.10, 5.20	8 (3.14)	2.13	1.00, 4.56	46 (3.11)	2.11	1.40, 3.18
**Hychol**	766 (24.41)	**442 (64.06)**	5.52	4.63, 6.58	**190 (74.51)**	9.05	6.75, 12.14	**933 (63.00)**	5.27	4.61, 6.02
**Heart attack**	42 (1.34)	99 (14.35)	12.35	8.52, 17.90	38 (14.90)	12.91	8.15, 20.44	126 (8.51)	6.85	4.81, 9.78
**CVD**	36 (1.15)	127 (18.41)	19.44[b]	13.28, 28.45	54 (21.18)	23.15[b]	14.83, 36.13	169 (11.41)	11.10	7.70, 16.00
**Total**	3138	690			255			1481		

**P2**										
**T2D**	136 (5.70)	163 (33.61)	8.45	6.54, 10.92	**423 (94.00)**	258.27[b]	168.71, 395.36	260 (11.59)	2.17	1.75, 2.70
**HTN**	687 (28.79)	**395 (81.44)**	11.3	8.81, 14.51	**353 (78.44)**	8.96	7.04, 11.41	**1461(65.14)**	4.66	4.11, 5.28
**Stroke**	33 (1.38)	37 (7.63)	5.92	3.66, 9.57	19 (4.22)	3.13	1.77, 5.56	101 (4.50)	3.37	2.26, 5.01
**Hychol**	565 (23.68)	**361 (74.43)**	9.65	7.69, 12.11	**333 (74.00)**	9.13	7.25, 11.50	**1264 (56.35)**	4.19	3.69, 4.75
**Heart attack**	50 (2.10)	159 (32.78)	22.99[b]	16.39, 32.25	37 (8.22)	4.17	2.69, 6.46	123 (5.48)	2.71	1.94, 3.79
**CVD**	50 (2.10)	200 (41.24)	33.14[b]	23.74, 46.27	48 (10.67)	5.56	3.69, 8.38	209 (9.32)	4.81	3.51, 6.58
**Total**	2386	485			450			2243		

CVD: cardiovascular disease, CI: confidence interval, HTN: hypertension, LC: latent class, P1: phase 1, P2: phase 2 (5-year follow up), OR: odds ratio, T2d: Type 2 diabetes.

a. Comparison of self-report comorbidity among each LC was conducted through unadjusted logistic regression. Low medication is the reference group. The bold numbers under n (%) showed high correlation (>50%) between medication use and self-report disease.

b. The large odds ratios (OR) were caused by either having very small percentage of people who reported "Yes" in the reference group (<5%) or having large percentage of "Yes" in the group of interest (>85%). OR>1 means the chronic disease condition was predicted by the treatment latent class (the assessed medication pattern had higher odds of self-report comorbidity compared to the low medication group). Larger OR shows the assessed medication pattern significantly predicts the self-report comorbidity compared to the low medication group, which shows medication patterns are indications of disease patterns.

Table S4 Death rate based on previous published P1 medication patterns

Previous LCA groups [a]	Death at 5- year follow up	Total number	Death rate
LCA 0	479	5234	0.09
LCA 1	82	648	0.13
LCA 2	36	362	0.10
LCA 3	127	969	0.13
LCA 4	23	123	0.19
LCA 5	119	1035	0.11
LCA 6	62	342	0.18
LCA 7	206	1414	0.15
Total	1134	10127	0.11

LCA: latent class analysis, P1: phase 1.

a.LCA 0-LCA 7 were medication patterns published previously based on a total of 10,127 participants who had phase 1 medication data in the COPDGene cohort. LCA 0: Low medication, LCA 1: angiotensin II receptor blockers (ARB)+statin, LCA 2: angiotensin-converting enzyme inhibitors (ACEi)+Statin+ type 2 diabetes medication (T2D), LCA 3: HTN+APT+Statin, LCA 4: hypertension medication (HTN)+T2D+statin+antiplatelet (APT), LCA 5: selective serotonin reuptake inhibitors (SSRI) only, LCA 6: HTN+SSRI+statin+APT, LCA 7: ACEi+diuretic.

Table S5 Comparison of P1 demographics between included and excluded participants

**P1 Characteristics**		**Inclusion in study cohort (n=5,564)**		**Exclusion (n=4,634)**		***P*-value [b]**
		**n (%)**	**Mean (SD)**	**n (%)**	**Mean (SD)**	
Age			60.09 (8.68)		58.81 (9.42)	0.33
Female		2780 (49.96)			1966 (42.43)	0.001
African American		1584 (28.47)				0.63
Smoking status	Former smoker	2957 (53.15)		1825 (39.38)		0.16
	Current smoker	2607 (46.85)		2809 (60.62)		
Smoking pack-years			42.89 (23.62)		45.70 (26.35)	0.20
BMI			29.09 (6.11)		28.52 (6.46)	<0.0001
COPD GOLD [a]	PRISm	689 (12.38)		573 (12.54)		0.0003
	GOLD 0	2541 (45.67)		1846 (40.41)		
	GOLD 1	492 (8.84)		295 (6.46)		
	GOLD 2	1122 (20.17)		804 (17.60)		
	GOLD 3	570 (10.24)		594 (13.00)		
	GOLD 4	150 (2.70)		456 (9.98)		

BMI: body mass index, COPD GOLD: global initiative for chronic obstructive pulmonary disease, P1: phase 1, P2: phase 2 (5-year follow up), PRISm: preserved ratio impaired spirometry, SD: standard deviation

a. The total number is not 5,564 because of missing data.

b. Comparison of mean of smoking pack-years between inclusion and exclusion used t-test. Comparison of mean of age and BMI between inclusion and exclusion used Wilcoxon test because of unequal variance. Comparison of

frequencies of gender, smoking status, COPD GOLD used chi-square test. The excluded population has less percentage of female, lower average BMI, higher percentage of GOLD 3 and GOLD 4 participants.
